# Fox sign in a case of terminal stage pancreatic cancer and suggestions for diagnosis

**DOI:** 10.1007/s12024-021-00392-y

**Published:** 2021-06-30

**Authors:** Julian Prangenberg, Elke Doberentz, Burkhard Madea

**Affiliations:** Institute of Legal Medicine, University Hospital Bonn, Stiftsplatz 12, 53111 Bonn, Germany

**Keywords:** Skin sign associated with pancreatitis, Pancreatitis, Fox’s sign, Grey Turner’s sign, Immunohistochemistry

## Abstract

Skin signs in acute pancreatitis are well-known and frequently discussed manifestations accompanied by unfavorable prognoses although they may rarely appear in clinical and forensic medicine. In 2018, the district attorney’s office ordered a forensic autopsy for a 74-year-old man with terminal stage pancreatic cancer. The autopsy was ordered based on accusations of the deceased’s widow regarding alleged medical malpractice and poor hospital care. The widow filed a grievance about multiple unsuccessful attempts to draw blood from her husband in addition to a diaper dermatitis at the right groin. An autopsy and additional histological examinations were performed. After considering all findings, the diaper dermatitis was eventually assumed to be a Fox sign caused by acute pancreatitis, and the allegations of medical malpractice were refuted. This case led us to identify another case with suspected cutaneous manifestations in pancreatic disease. We performed immunohistochemical staining on those two cases and six control cases to examine whether there was detectable presence of pancreatic lipase and trypsin in the skin discolorations and whether it could be used as a feasible method to verify skin signs associated with pancreatitis. Based on our findings, a minor disseminated lipase and trypsin staining should be considered regular and is therefore not conclusive of a skin sign associated with pancreatitis. Moreover, trypsin does not seem to be as suitable as lipase for this suggested immunohistochemical method. Nevertheless, this method might be a useful addition for determining the origin of skin discoloration and verifying skin signs associated with pancreatitis.

## Introduction

Dermatologic findings, which are frequently observed during autopsies, can provide valuable differential diagnostic clues regarding their underlying etiology. Such findings sometimes even indicate cause of death [1, 2]. These findings include various skin signs in acute pancreatitis that are defined based on their particular expansion and location of emergence. A commonly accepted view on pancreatitis is that the premature activation of digestive enzymes within the pancreas leads to autodigestion of the organ [3–5]. The enzymes include proteases such as trypsin, elastase, and pancreatic lipase [6]. Skin signs associated with pancreatitis are presumably caused by the expansion of intra- and retroperitoneal hemorrhages along anatomic structures, which manifest in the subcutaneous tissue at specific locations, depending on the path of expansion [7]. However, these signs do not solely occur in acute pancreatitis, and many other causes have been identified, including necrotizing pancreatitis, intraperitoneal hemorrhage, portal hypertension, liver cirrhosis anticoagulation with warfarin, or deep vein thrombosis [8–13]. Furthermore, they indicate a poor prognosis [14]; therefore, it seems reasonable to raise awareness regarding the potential clinical impact they entail. Grey Turner’s sign [15] and Cullen’s sign [16] represent the best-known signs and are frequently mentioned in textbooks and journals. A lesser-known skin sign is the Fox sign, which emerges from the extraperitoneal expansion of pancreatic fluid along the fascia of the psoas and iliacus until its subcutaneous manifestation below the inguinal ligament [17]. By contrast, the Fox sign is rather rare in clinical practice, and some physicians may have never encountered such a case themselves.

In 2018, we encountered an autopsy that was ordered by the district attorney’s office owing to alleged medical malpractice and poor hospital care in which the Fox sign was assumed to be present and later used to refute the raised allegations. Furthermore, we identified another case with skin signs associated with suspected pancreatitis and examined how to identify skin signs in suspected pancreatitis using immunohistochemistry.

## Methods

Two forensic autopsy cases, in which the deceased exhibited skin discolorations at typical locations for skin signs in pancreatitis at the external examination, were identified between 2018 and 2019. The presence of those skin signs and an additional pancreatic disease was the inclusion criterion. Skin samples of the discolorations were obtained, and histologic examinations and immunohistochemical staining were performed. Six autopsy cases served as controls. Five controls had no skin discolorations or relevant organ pathologies; one case showed a reddish-livid discoloration at the right flank but no pancreatic pathologies. The cause of death was hanging in three cases and gunshot to the head, severe head injury, and myocardial infarction in one case each.

The immunohistochemical staining used the following antibodies: rabbit monoclonal anti-pancreatic lipase antibody (EPR6276) ab124915 and rabbit monoclonal anti-trypsin antibody (EPR19498-15) ab211491 (Abcam, Cambridge, United Kingdom). In accordance with the manufacturer’s instructions, the immune complexes were visualized using the Dako Envision + Dual Link System (Dako Denmark, Glostrup, Denmark). After staining and preparation, the slides were microscopically examined using the Olympus BX 53 photomicroscope with the Olympus SC 180 camera (Olympus Corporation, Tokyo, Japan) with a sensor size of 4.8 × 2.7 mm at an eyepiece magnification of 10 × and an objective magnification between 4 × and 20 × .

### Case 1

The district attorney’s office ordered a forensic autopsy of a 74-year-old male owing to allegations regarding medical malpractice. The patient had a known pancreatic carcinoma and was taken to the hospital for angina and dyspnea. During hospitalization, a pulmonary embolism and hepatic metastases were diagnosed. The patient was also treated with an unspecified anticoagulating agent owing to cardiac arrhythmia. Despite curative treatment, his medical condition deteriorated, and he died a few days after admission. The clinical cause of death was stated as a metastasized pancreatic carcinoma with hepatic failure. The widow filed a grievance about the treatment, including multiple unsuccessful attempts to draw blood from her husband during his in-patient stay and diaper dermatitis at the right groin due to alleged poor hospital care.

**Fig. 1:**
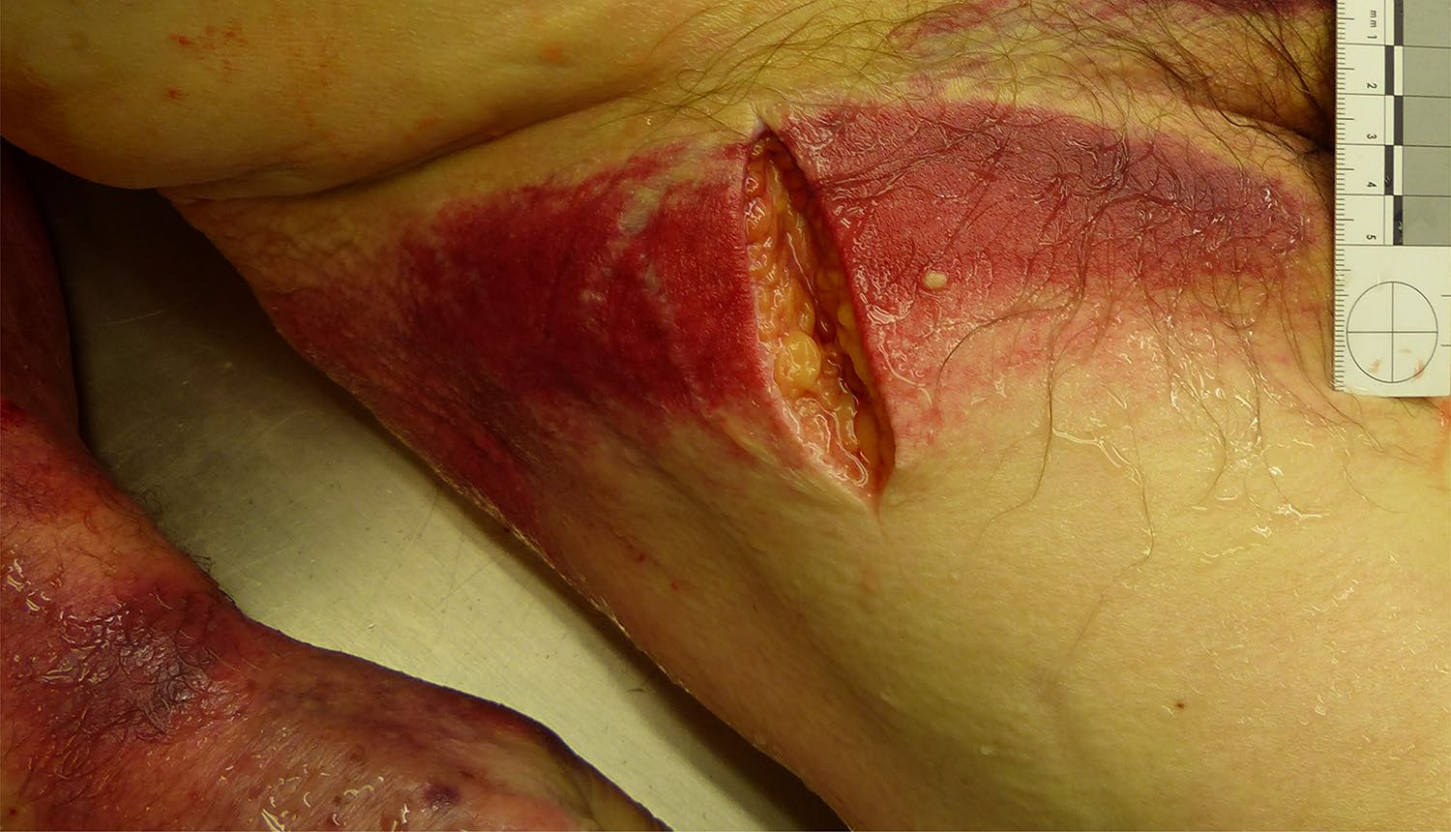
Suspected Fox sign at the right groin (case 1)

External examination of the 75-year-old male (96 kg, 175 cm) showed distinctive anemia and pale skin and mucosa. Both arms and the right groin displayed extensive reddish-livid discoloration (Fig. 1). Internal examination revealed a progressed pancreatic carcinoma with multiple metastases in the liver, lungs, lymph nodes, and abdominal wall. Furthermore, the autopsy identified a thyroid tumor, a space-consuming lesion in the mediastinum, an exceeding of the critical heart weight (660 g), renal infarctions and shock-kidneys, old cerebral infarctions, and cystitis. No signs of medical malpractice were found, and the nursing care was evaluated as unobjectionable. The assumed diaper dermatitis was suspected to be a Fox sign due to acute or necrotizing pancreatitis. The cause of death was eventually defined as a metastasized terminal stage pancreatic carcinoma.

Additional histological investigations were performed to confirm the presumption of the Fox sign. Histological investigations identified necrotizing pancreatitis due to a pancreatic adenocarcinoma. The skin sample of the right groin revealed an edema of the subcutaneous tissue flushed with intact erythrocytes, fatty tissue necrosis, and slight perivascular infiltration of lymphocytes. An acanthosis or spongiosis as an indicator of underlying dermatitis was not detected. Immunohistochemical staining of trypsin and pancreatic lipase in the skin sample was performed to substantiate the assumption of the Fox sign. Immunohistochemical analysis did not show any reddish staining for trypsin and little disseminated staining for pancreatic lipase in the dermis.

### Case 2

A 46-year-old male (55 kg, 167 cm) complained of abdominal pain and blood in his vomit the past few days. He initially refused admission to the hospital. The paramedics were then called to his apartment, but he died after unsuccessful resuscitation in his apartment.

**Fig. 2:**
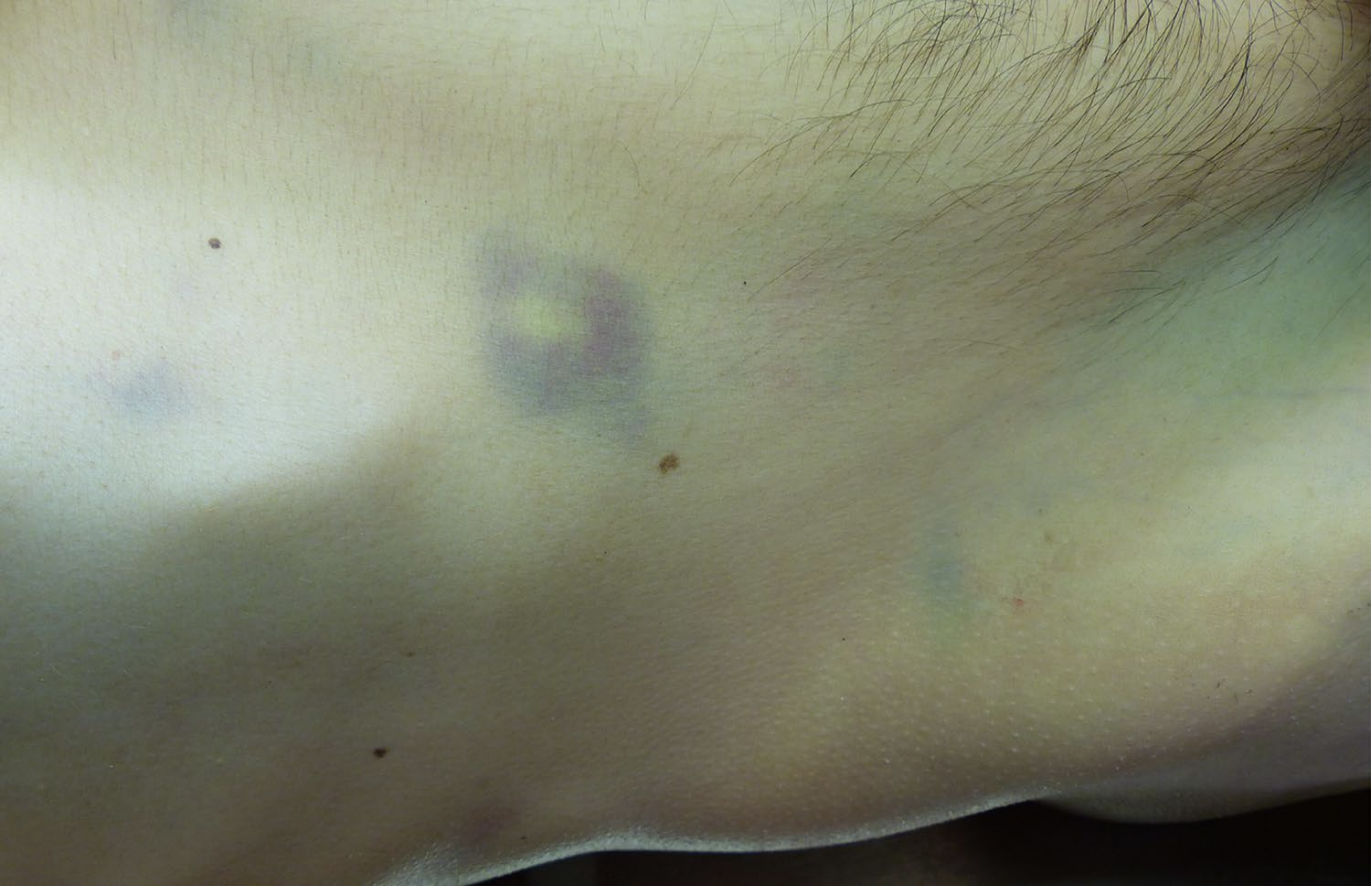
Livid discoloration at the right flank (case 3)

External examination showed a pale, bluish discoloration with central yellowish staining over his right costal arch (Fig. 2). Forensic autopsy revealed a cachectic constitution, a fatty liver with beginning cirrhosis, a necrotic and inflamed pancreatic head with surrounding hemorrhage, and hemorrhage in the right-sided mesocolon and right renal pelvis. The cause of death was determined to be necrotizing pancreatitis with internal bleeding.

**Fig. 3:**
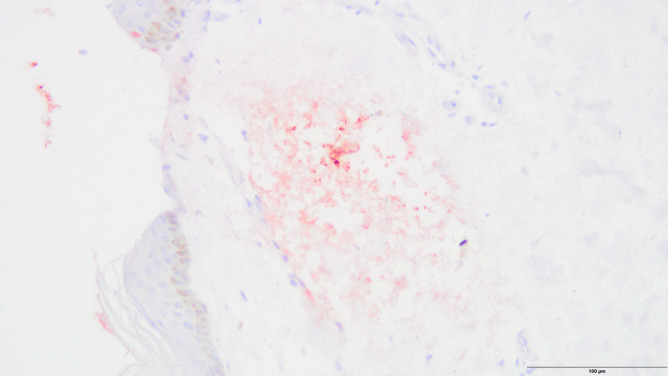
Extensive reddish staining of lipase in the epidermis and dermis (case 3, anti-pancreatic lipase antibody, 200 ×)

**Fig. 4:**
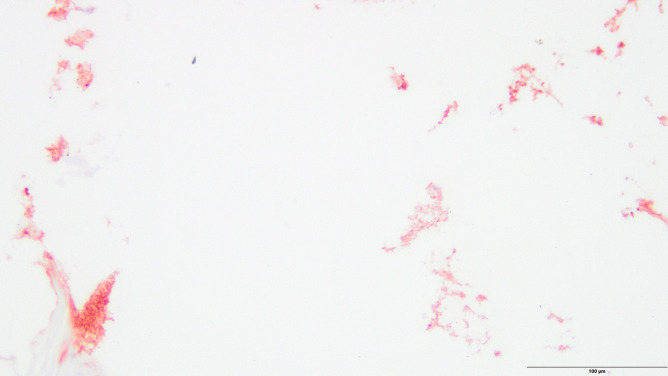
Extensive reddish staining of lipase in the epidermis and subcutaneous tissue (case 3, anti-pancreatic lipase antibody, 200 ×)

**Fig. 5:**
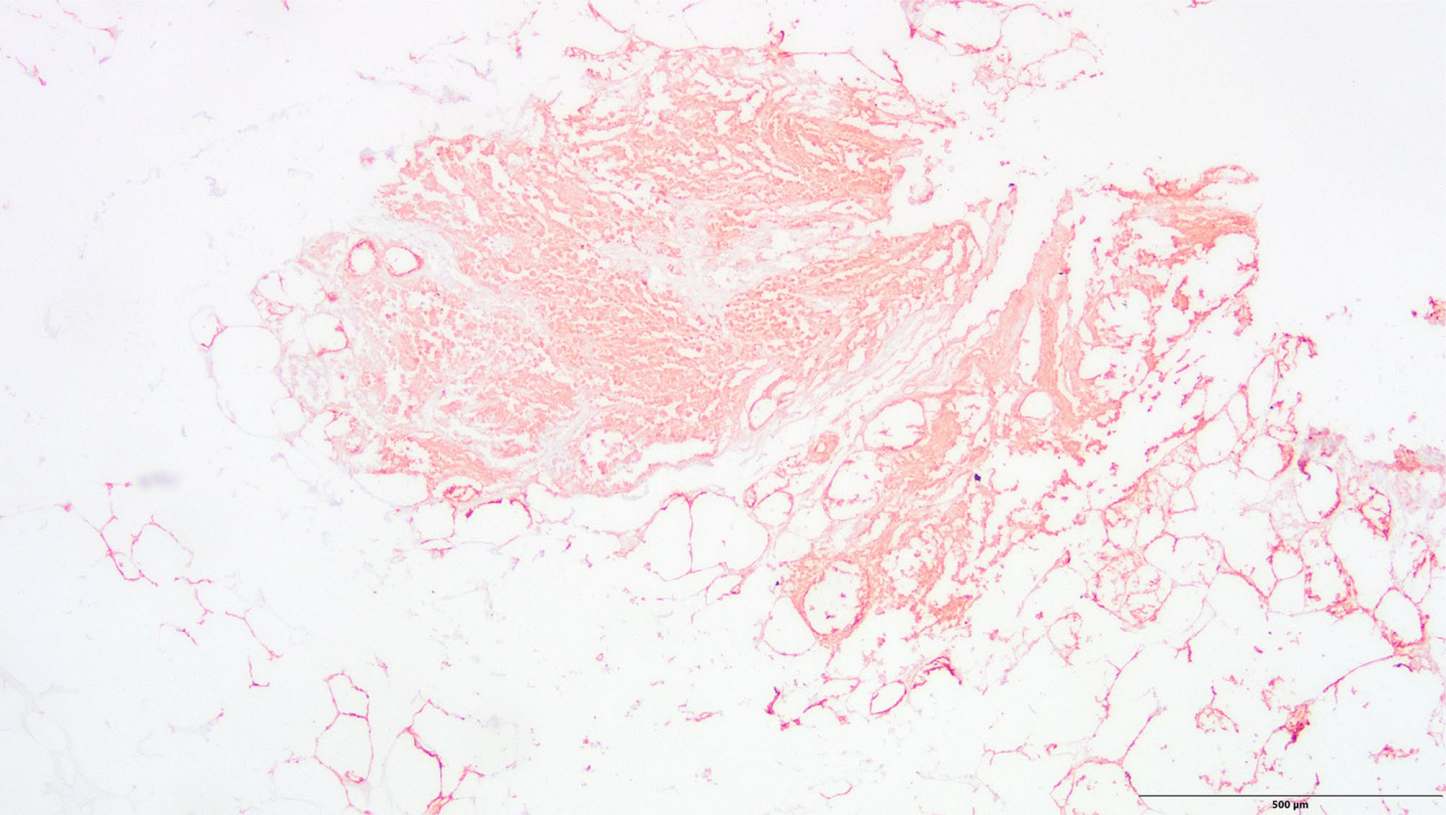
Extensive widespread staining of lipase in the pancreas due to necrotizing pancreatitis (case 3, anti-pancreatic lipase antibody, 40 ×)

Immunohistochemistry did not show any signs of reddish staining for trypsin; however, there was extensive staining for pancreatic lipase in the epidermis and dermis and widespread staining throughout the pancreas owing to necrotizing pancreatitis (Figs. 3, 4, 5).

### Control cases

Macroscopically uninjured abdominal skin of five males aged between 43 and 66 years (mean, 52.4 years) and the skin discoloration of an 89-year-old female with an initially suspected Grey Turner’s sign were examined. No relevant organ pathologies or other factors described in the current literature that potentially cause skin signs were present. The cause of death was hanging in three cases, gunshot to the head in one case, and severe head injury in one case.

**Fig. 6:**
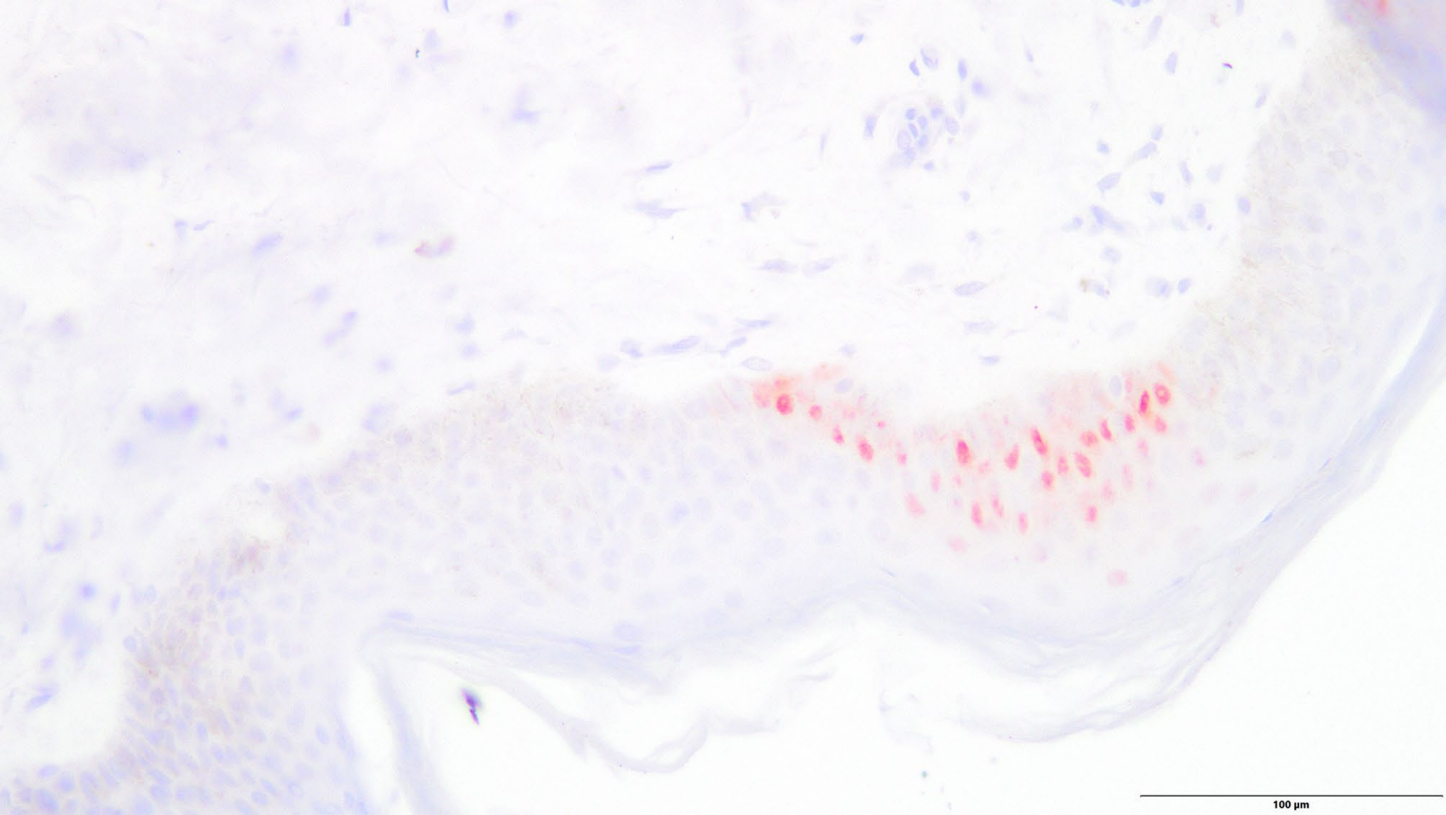
Circumscribed reddish staining of trypsin in the epidermis (control case, anti-trypsin antibody, 200 ×)

**Fig. 7:**
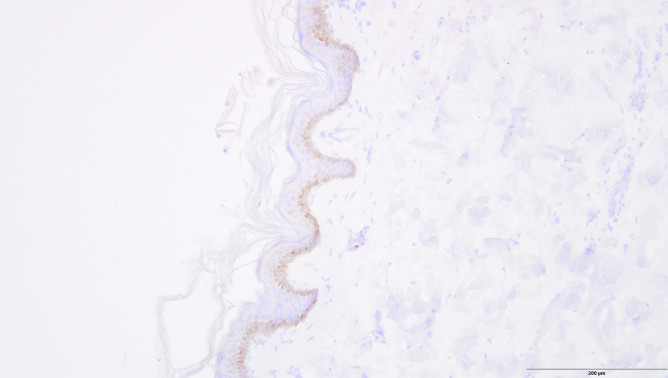
Disseminated reddish staining of trypsin in the epidermis (control case, anti-pancreatic lipase antibody, 200 ×)

Immunohistochemical analysis occasionally showed very little disseminated staining for pancreatic lipase in the dermis, but there was no extensive staining as shown in case 2. One case displayed moderate circumscribed staining in the epidermis for trypsin (Figs. 6 and 7).

## Discussion

In forensic analyses, determining the origin and etiology of skin discoloration to exclude or confirm bodily harm can be crucial. This, among other factors, requires knowledge of the described skin signs in pancreatitis and other conditions that may cause these specific discolorations. The literature describes certain conditions that potentially cause the Fox sign, such as necrotizing pancreatitis, deep vein thrombosis, and rupture of an abdominal aortic aneurysm [8, 13, 17].

In case 1, a pulmonary embolism was diagnosed in the hospital before the man’s death. Because pancreatic carcinoma has the highest risk for the development of vein thrombosis among other carcinomas [18] and approximately 93% of thromboses originate in the inferior vena cava and pelvic and leg veins [19], a deep vein thrombosis could also have caused the Fox sign. There were no signs of inflammation indicating a diaper dermatitis in the skin. Immunohistochemical analysis showed a low level of staining for pancreatic lipase in the subcutaneous skin and no staining for trypsin. However, the pancreatic lipase staining was slightly more distinct than in the control cases, and after considering all factors, the discoloration was assumed to be a Fox sign. To the best of our knowledge, identifying a presumed diaper dermatitis as a Fox sign and thus refuting allegations of medical malpractice and poor hospital care appear to represent a novelty. Furthermore, this case emphasizes the importance and explanatory power of forensic autopsies and complementary histological examinations regarding alleged medical malpractice.

The male in case 2 had a fatty liver with the beginning of cirrhosis, a necrotic and inflamed pancreatic head with surrounding hemorrhage, and hemorrhage in the right-sided mesocolon and right renal pelvis. The cause of death was determined as necrotizing pancreatitis with internal bleeding. There were no signs of reddish staining for trypsin; however, there was extensive staining for pancreatic lipase in the epidermis and dermis that was much more intense than in any of the included cases. Considering the immunohistochemical results and because the person had two conditions that are known to cause such discoloration, Grey Turner’s sign was assumed to be present in this case.

Overall, the differential diagnostics of skin discolorations should include the distinction from hematoma or ecchymosis due to blunt force or needle puncture sites because this could feign the origin of skin signs associated with pancreatitis [20]. In both cases, there was no clear evidence of blunt force or needle punctures although small injections are not always visible and therefore cannot be ruled out with absolute certainty.

Regarding the control group, one case showed circumscribed staining of trypsin in the epidermis and very slight disseminated staining of lipase in the dermis. This might be owing to cross-reactions with the trypsin antibody and other serine proteases present in human skin [21]. Talas et al. [22] examined the expression of human elastase 1 in the skin that showed results similar to those found in both our study and control cases. Lipase can be detected in the sebaceous glands and in the external root sheath of the hair follicle [23]. Therefore, lipase enzymes were believed to be dissolved during staining, which resulted in their detection in normal skin. Skin discoloration at the right flank observed in the 89-year-old female may be explained by the use of anticoagulants. The use of an anticoagulating agent with warfarin is described in the literature to form skin discoloration at this location [12]. Unfortunately, no information on anticoagulating agents or other medication was available. Thus, the presence of Grey Turner´s sign was considered unlikely and the discoloration presumably had a different cause.

Based on our findings, minor disseminated staining of lipase and trypsin should be considered regular and alone should not lead to the conclusion of a skin sign associated with pancreatitis. Considering all cases, trypsin does not seem as suitable as lipase for this suggested immunohistochemical method. Furthermore, in the three cases with skin signs associated with suspected pancreatitis, it remained unclear when the discoloration first occurred; therefore, both the antemortem and postmortem degradation of the enzymes must be considered, which might have led to the indistinct staining pattern in two cases.

Conclusively, additional immunohistochemical investigations regarding the presence of pancreatic lipase may be useful in cases with unclear skin discoloration. However, considering the relative rarity of this phenomenon, further and ideally multicenter studies with a sufficient number of cases are warranted to establish and verify reliable staining patterns for the examined enzymes.

## Key Points:

1. Dermatologic findings are frequently observed during autopsies and can provide valuable information.
2. A variety of conditions can cause skin discoloration in specific locations.
3. We performed immunohistochemical investigations to prove the presence of pancreatic enzymes in the skin.
4. Trypsin does not appear to be a suitable marker for verifying skin signs associated with pancreatitis.
5. Pancreatic lipase may be a useful marker for determining the etiology of skin discoloration.
